# MyopiaDETR: End-to-end pathological myopia detection based on transformer using 2D fundus images

**DOI:** 10.3389/fnins.2023.1130609

**Published:** 2023-02-07

**Authors:** Manyu Li, Shichang Liu, Zihan Wang, Xin Li, Zezhong Yan, Renping Zhu, Zhijiang Wan

**Affiliations:** ^1^School of Information Engineering, Nanchang University, Jiangxi, China; ^2^School of Computer Science, Shaanxi Normal University, Xi’an, China; ^3^Industrial Institute of Artificial Intelligence, Nanchang University, Jiangxi, China; ^4^School of Information Management, Wuhan University, Hubei, China

**Keywords:** myopia detection, fundus images, attentional FPN, detection transformer (DETR), dichotomous graph matching

## Abstract

**Background:**

Automated diagnosis of various retinal diseases based on fundus images can serve as an important clinical decision aid for curing vision loss. However, developing such an automated diagnostic solution is challenged by the characteristics of lesion area in 2D fundus images, such as morphology irregularity, imaging angle, and insufficient data.

**Methods:**

To overcome those challenges, we propose a novel deep learning model named MyopiaDETR to detect the lesion area of normal myopia (NM), high myopia (HM) and pathological myopia (PM) using 2D fundus images provided by the iChallenge-PM dataset. To solve the challenge of morphology irregularity, we present a novel attentional FPN architecture and generate multi-scale feature maps to a traditional Detection Transformer (DETR) for detecting irregular lesion more accurate. Then, we choose the DETR structure to view the lesion from the perspective of set prediction and capture better global information. Several data augmentation methods are used on the iChallenge-PM dataset to solve the challenge of insufficient data.

**Results:**

The experimental results demonstrate that our model achieves excellent localization and classification performance on the iChallenge-PM dataset, reaching AP_50_ of 86.32%.

**Conclusion:**

Our model is effective to detect lesion areas in 2D fundus images. The model not only achieves a significant improvement in capturing small objects, but also a significant improvement in convergence speed during training.

## Introduction

Retinal diseases are one of the main causes of vision loss, and severe retinal diseases can also cause irreversible damage to vision. Medical research has found that the deformation of the front of the eyeball varies with the degree of myopia (Wong et al., 2014), these changes may be related to the complications of ocular diseases, the complications of pathological myopia (PM) are considered to be the main cause of visual impairment and blindness. Due to changes in the environment and lifestyle, the incidence of high myopia-related diseases has been increasing year by year (Hsu et al., 2004; Iwase et al., 2006; Yamada et al., 2010; You et al., 2011; Furtado et al., 2012). As a common eye disease, it affects 20 to 40% of adults (Iwase et al., 2006) and has become a global burden of public health, 35% of myopic patients are high myopia (HM) (Yamada et al., 2010), which will develop into pathological myopia. The PM is characterized by excessive and progressive elongation of the globe, which is now considered to be the most visually impaired and blind cause. Therefore, timely diagnosis and regular review for PM are very important.

Nowadays, people pay more attention to their health, and the demand for medical services is also increasing. Although the number of ophthalmologists in the developed countries is growing (Hsu et al., 2004; You et al., 2011; Furtado et al., 2012; Sakaguchi et al., 2019), there is still a big gap in the demand for ophthalmologists. Due to the long training time for cultivating doctors, the underdeveloped regions will still face the problem of shortage of medical resources in the next few decades. With the development of imaging technology, myopia-related complications have been identified (Lu et al., 2018; Peng et al., 2019; Nazir et al., 2020; Cui et al., 2021; Zhang et al., 2021; Muthukannan and Glaret Subin, 2022). At present, fundus imaging is an important basis for the diagnosis of various ophthalmic diseases. Most retinal diseases can be avoided with early and timely treatment. Therefore, early detection and early treatment are of great significance for the cure of retinal diseases. However, analysing medical images relies on the extensive medical experience of doctors, which is laborious and time-consuming. Thus, designing a reliable and accurate automatic detection method for fundus images is crucial to the prevention and treatment of diseases.

Many studies utilize deep learning techniques to diagnose eye diseases. For instance, Nazir et al. (2020) proposed a FRCNN algorithm with fuzzy k-means for automatic detecting three types of retinal diseases at early stage. Vyas et al. used common convolutional neural network for dry eye disease (DED) detection based on Tear Film Breakup Time (TBUT) videos, the approach shows high performance in classifying TBUT frames and detecting DED. Muthukannan and Glaret Subin (2022) designed a CNN that optimized by flower pollination for feature extraction, increased the speed and the accuracy of the network for detecting four types of eye diseases. However, most efforts in the existed deep learning focused on applying existing techniques to the myopia detection task rather than proposing new ones specifically suited to the domain. The standard well-known network architectures were designed for the data collected in natural scenes (e.g., natural images) and do not take the peculiarities of the myopia images’ characteristics into account. Therefore, research is necessary to understand how these architectures can be optimized for myopia data.

Detection Transformer (DETR) is a new paradigm for end-to-end object detection. DETR always failed in detecting small object and it has a slow convergence speed. Since DETR only utilizes the feature maps (32 × down sampling) from the last layer of backbone, which leads to a large semantic loss of small objects, thus DETR performs poorly on small object detection. Additionally, in the decoder structure of DETR, self-attention is computed for all input pixels, so the model is presented with computational complexity in square level, which further results in slow convergence speed. To solve the problems of the two aspects, Deformable DETR (Zhu et al., 2020) improves the performance of small object detection and accelerates the convergence speed by limiting the range of computed attention using multi-scale feature maps. Conditional DETR (Meng et al., 2021) uses the conditional spatial query explicitly to find the extremity region of the object to reduce the searching range of object and accelerate the convergence.

**FIGURE 1:**
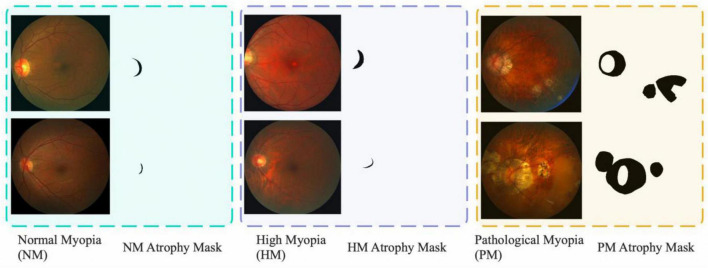
Fundus images selected from iChallenge-PM dataset. The dataset contains three types of 2D fundus images (NM, HM, PM).

Figure 1 shows some examples selected from the iChallenge-PM dataset. The figure shows the typical image characteristics of the fundus images, the green background indicates normal myopia (NM), the purple background represents high myopia (HM), and the yellow background delegates pathological myopia (PM). The black mask on the right side of myopia image is atrophy area, which various a lot in shape or to some extend very similar. The white oval area in yellow background is eye’s optical disk region and the lesion area appears randomly. These characteristics challenge the model performance of the deep learning methods. The specific challenges are illustrated as follows:

(1) **Morphology Irregularity:** As for the NM and HM shown in Figure 1, the lesion area which in green and purple background is irregular and similar, its area only occupies a small part of the location, which makes the model troubling to learn its morphological features and the most of the rest area is background.

(2) **Imaging Angle:** From all images in Figure 1, it is obvious that the optical disk region hava a tendency to the left of the image, this man-made imaging method may mislead our model, so the differences brought by the imaging angle require the learning ability of the model for location correlation demanding.

(3) **Insufficient Data:** The iChallenge-PM dataset only contains 1,200 images, around 600 images for PM and 600 images for Non-PM (NM+HM). Fewer images and the strong fitting ability of neural network make it easy to overfit, which will reduce the generalization ability of the model.

In this paper, a novel deep learning model named MyopiaDETR is proposed for detecting the lesion of NM, HM, and PM using 2D fundus images. Our base model adopts scalable Swin Transformer as backbone, which is flexible in depth. When it comes to morphology irregularity, it is worth noting that the lesion tissue is not only in irregular shape, but also distributed in a small area of the whole fundus image, most of the pixels are redundant, and an impure background will adversely affect the prediction results. To address those problems, we propose a novel Attentional feature pyramid networks (FPN) architecture that can purify the feature representation during the aggregation of feature maps, specifically, object queries are added to FPN (Lin et al., 2017) levels with positional encoding to execute multi-head self-attention, which are used to give more activation weight to the object regions, produce a larger gap between object and background. Our attentional FPN solves the problem that traditional DETR cannot utilize multi-scale feature maps, resulting in poor performance in detecting small objects in the fundus image. Since most of the regions in the fundus image are background or useless regions, we want our model to focus on the ophthalmic disease regions of interest so as to reduce the computational complexity.

As for the imaging angle, unlike the traditional object detection method, which first generates many candidate boxes on the image, and then adjusts the offset of the boxes according to the calculated loss between the predicted results and the real labels. This method has strong traces of artificial design, which is different from the way humans observe objects, and must require post-processing methods like Non-maximum suppression (NMS) (Neubeck and Van Gool, 2006), whose drawbacks are inevitable in the face of large overlapping areas between real labels. The DETR (Carion et al., 2020) structure, on the other hand, views the object detection from the perspective of set prediction, calculates the loss through the dichotomous graph matching method, and the Transformer structure has global information, which is more in line with the way humans observe objects. Therefore, we choose the DETR structure for further optimization in the face of sensitive location information.

Finally for the insufficient data mentioned above, Ghiasi et al. (2021) used data augmentation to address the insufficiency of the training data for classifying PM. Almahairi et al. (2018) used CycleGAN with cycle consistency to generate more realistic and reliable images for training, a res-guided U-Net is constructed for segmentation, they achieved superior result on PM detection. We adopt variety of strong data augmentation to enrich iChallenge-PM dataset while training our model. Unlike the common two-branch head design, inspired by DETR, we use a feed forward network that takes the output from Transformer decoder, which will produce the box coordinates and a matrix for classification if the query has an object, no post-processing is needed.

The main contributions of this paper are summarized as follows:

(1) Inspired by DETR, we present a novel post-processing free object detector that uses fundus image data for pathological myopia diagnosis. For specific, we design an attentional FPN that uses object queries on feature maps of each level of FPN, the self-attention mechanism increases the feature intensity gap between foreground and background. Due to the architecture of DETR, it can well solve the challenge of morphology irregularity. To the best of our knowledge, this is the first work using object detection for pathological myopia diagnosis based on iChallenge-PM dataset.

(2) Several data augmentation methods are used on the iChallenge-PM dataset to accelerate model convergence and enhance model robustness.

(3) Extensive experiments are conducted on iChallenge-PM dataset for discriminating NM, HM, and PM. The results demonstrate the superiority of our method than other state-of-the-art (SOTA) object detectors.

The rest of this paper is organized as follows. Section “Related works” introduces related works of the deep learning based retinal disease analysis methods. Section “Materials and methods” illustrates the details of our proposed MyopiaDETR model, which comprizes of Swin Transformer (Liu et al., 2021) backbone, attentional FPN, Transformer encoder, and decoder, shared feed forward network for specific retinal disease analysis tasks. Section “Experimental results” describes the experimental results of ablation studies and comparison studies. Finally, section “Discussion” has a discussion about our method and section “Conclusion” presents the conclusions of this paper and expounds ideas of future work.

## Related works

### Deep learning based object detection

Object detection is a popular task in computer vision and is widely applied in many real-world scenes such as autonomous driving, video surveillance, remote sensing, and medical diagnosis. The main task of object detection is to locate and classify the target of interest from an image. In the context of the rapid development of computing power, deep learning has been researched and applied as never before. In the trajectory of vision model, AlexNet (Krizhevsky et al., 2017) opened a new era of computer vision by using convolutional neural network years ago, making CNN architecture the mainstream approach of deep learning for many years. Object detectors can be divided into anchor-based and anchor-free model based on whether or not anchor is used during the detection pipeline. The anchor based models can be further divided into two-stage and one-stage detector. One-stage model predicts bounding boxes on grid while two-stage model uses a proposal network to generate candidate boxes, and then uses a second network to refine the result. The advantage of one-stage detectors is that it can complete localization and classification by going through the network once, hence the one-stage detectors can offer significant advantages in terms of speed, such as SSD (Liu et al., 2016) and YOLO (Redmon et al., 2016; Redmon and Farhadi, 2017, 2018; Bochkovskiy et al., 2020) series. Two-stage models sacrifice speed for obtaining high accuracy, most of the mainstream detectors with high performance adopt two-stage methods, such as Faster R-CNN (Ren et al., 2015) and Cascade R-CNN (Cai and Vasconcelos, 2018). With the trend of Transformer architecture gradually unifying natural language processing (NLP) and computer vision (CV), Vision Transformer (Dosovitskiy et al., 2020) (ViT) are gradually dominating visual tasks. The excellent relational modeling capability of self-attention mechanism is bringing feature extraction to a new era. Pyramid Vision Transformer brought pyramid structure in to Transformer, making it seamlessly accessible to a variety of downstream tasks (e.g., object detection, semantic segmentation). Swin Transformer (Swin-T) proposed a vision transformer with sliding window operation and hierarchical design, achieved state-of-the-art in many tasks. The hierarchical design makes feature fusion easier. Swin Transformer is an improved version based on ViT, which has a similar structure of CNN. The hierarchical structure of Swin Transformer is more suitable to be applied to many downstream tasks. While ViT is a straight structure, it does not change the dimension of input feature map. In addition, the resolution of fundus image is generally large, and many rich semantic features will be lost if the image shape is resized to a range that is acceptable to ViT. Swin Transformer can receive larger image resolution, which means the Swin Transformer can do better in processing images with large resolution than ViT model. Thus, we choose Swin Transformer as our feature extraction backbone network.

### Deep learning based eye diseases detection

The object in medical images usually have small sizes and certain morphological features. Many studies utilize deep learning techniques to diagnose eye diseases. Early work such as (Liu et al., 2010) developed a system called PAMELA (Pathological Myopia Detection by Peripapillary Atrophy) that automatically identifies pathological myopia in retinal fundus images. (Wen et al., 2020) placed the key research on the distinction between pathological myopia and high myopia, a two-branch network is proposed, where the first branch distinguishes between normal and abnormal, while the other branch classifies pathological myopia and high myopia. Specifically, the previous studies on iChallenge-PM dataset have been related to image classification and instance segmentation. Cui et al. (2021) used data augmentation to address the insufficiency of the training data for classifying PM. Zhang et al. (2021) used CycleGAN with cycle consistency to generate more realistic and reliable images for training. A res-guided U-Net is also constructed for segmentation, they achieved superior result on PM detection. Our work first use an object detection method for classifying and locating the atrophy based on retinal fundus images. From the previous studies, we can know that the majority of automatic diagnose method uses CNN architecture for feature extraction, and to the best of our knowledge, our work is the first that uses Transformer architecture as backbone and also the first post-processing-free end-to-end detector in myopia diagnosis.

### Attention mechanism

With the trend of Transformer architecture gradually unifying NLP and CV (Gumbs et al., 2021), Vision Transformer is gradually dominating the visual tasks. In the evolution of vision attention mechanism, common method can be embedded into CNN for building more relevant feature, such as soft-attention. Soft-attention is a continuous distribution problem, focusing more on spatial or channel, it can be divided into spatial attention and channel attention. Non-local first utilized the idea of Transformer in computer vision model, it takes the approach of doing attention on the feature maps of the intermediate layers, which greatly avoids the computational cost. Self-attention based model, such as ViT and Swin Transformer, has excellent capabilities in extracting relationships between image patches, building the connections of those that are mostly relevant to each other. The pioneer work DETR is worth noting, it applies Transformer architecture as an end-to-end object detector, the post-processing-free design is more compatible with human visual patterns and also avoid the unstable performance of NMS. In the retinal fundus image scenario, the cluttered tissue makes it more difficult to extract the feature of atrophy, so we believe that the self-attention mechanism can help the model to better capture the difference between foreground and background.

## Materials and methods

Diagnosing myopia by detecting lesions based on fundus images requires sufficient data for the deep learning model to have a steady performance (Peng et al., 2019; Virmani et al., 2019). The iChallenge-PM dataset released by Baidu encourage the data-driven methods to automatically detect fundus lesion, it contains three types of fundus diseases images and lesion masks, as illustrate in Figure 1, previous studies try to design general deep learning based methods to address the fundus disease identification and localization problems, which contain image classification and segmentation that are insufficient and slow, respectively. In order to implement object detection on this dataset, we transform the mask of lesions into bounding box by obtaining the length and width of the mask. It can be clearly found from the sample images that the region occupied by the disease lesion is only a small part of the fundus image, and the rest of the tissue without lesions can be considered as redundant information, which will be detrimental to the feature extraction and representation of the model. That’s why we choose DETR as our baseline model, detail improvements are as follows.

### Overall architecture

**FIGURE 2:**
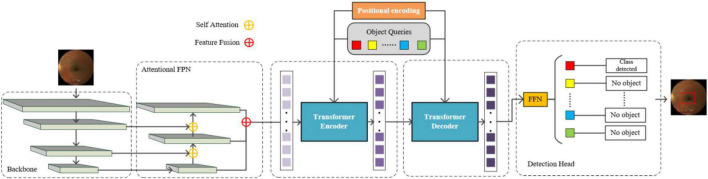
Overall architecture of our MyopiaDETR.

Inspired by DETR, the overall architecture is illustrated as Figure 2, the main components are: backbone network, attentional FPN, Transformer Encoder-Decoder, and a detection head, in which the backbone is responsible for feature extraction, the feature maps of three stages with different resolutions are fed into attentional FPN for feature aggregation. The outputs of attentional FPN are further sent to the encoder-decoder architecture, which consists of multi-head self-attention mechanism, layer normalization and a feed forward neural network, the details are similar to the DETR. Positional encoding is also adopted for retaining positional information of the feature blocks, while the object queries are used for information aggregation, give more attention to the positions where objects are likely to appear. The detection head is in charge of classifying the output of the decoder and finally getting the detection result. In particular, the yellow circles in the Figure 2 represent self-attention module, which calculates the similarity while fusing feature maps. The red circles represent multiple layers of features for fusion. Small squares of different colors in object queries represent different query objects. As the feature map flows from encoder to decoder, the feature representation becomes clearer and the purple squares become darker. The final feed forward network (FFN) is shared to calculate the object box position and category attributes of the object query. During the training phase, the image batch is fed into the backbone to obtain feature maps of four stages and reduce the computational overhead, three relatively small feature maps are selected and fed into the detection neck for integration. In the upsampling process of the neck network, self-attention operation is added to obtain features with better feature representation ability. Then the aggregation of the neck feature is sent to the following encoder part. In the detection head, FFN takes the output of decoder as input and utilizes bipartite matching loss (i.e., Hungarian Maximum Matching algorithms) to calculate the corresponding loss values. During inference phase, the learned object query generates box candidates through Transformer decoder to select boxes with larger confidence as the final prediction result.

### Model backbone

In the overall model architecture, the backbone is in charge of feature extraction of the image. The mainstream architecture of backbone are mainly divided into CNN [AlexNet, ResNet (He et al., 2016), Res2Net (Gao et al., 2019), ResNeXt (Xie et al., 2017), ConvNeXt (Liu et al., 2022)] and Transformer (e.g., ViT, Swin Transformer). The translational invariance and localization of CNNs provide inductive bias, makes CNN models converge faster, however, the fixed receptive field limits the global view of convolution operation. The positional encoding enables Transformer based network to obtain better capabilities in learning global dependencies. Swin Transformer first splits the image into small patches and then feed each patch as a token into Transformer encoder, the core idea is to calculate the similarity between patches for training an attention-intensive network without convolution operations. Compare with the Swin Transformer, the CNNs possess inductive bias, and their convergence speed is relatively fast. The advantages of inductive bias are reflected at two aspects: (1) the convolutional kernel size is generally fixed which result in high local correlation existed in feature maps; (2) the feature maps generated by the CNNs have characteristics of translational invariance, which indicates the output of the convolutional layer does not change no matter where the object appears in the image.

Since our neck network also uses the architecture of the self-attention mechanism, Swin Transformer is selected as our backbone so as to keep the consistency of the features representation. Moreover, the Transformer architecture has good parallel computing capability and global view characteristics. When we input a fundus image with the shape of H × W × C, it will first pass through a patch partition module with the purpose of descending and chunking the image. The output gets a sequence of N × (P2 × C) spreading 2D image blocks, where *N* is the number of image blocks, P2 is the area of each patch, H and W are the height and width of the image, respectively, and C is the number of image channels. Here we set P to 4, and *N* is computed by H/4 × W/4. In summary, an input image with the shape H × W × C passes through the patch partition module and outputs a tensor of H/4 × W/4 × 48, which can be understood as a total of H/4*W/4 image patches, each of which is composed of a 48-dimensional token. The W-MSA and SW-MSA modules help the Swin Transformer to improve its ability of extracting the global features in the fundus image. The W-MSA module restricts the receptive field of the model by only applying the self-attentive mechanism within each patch. The SW-MSA model adds a cyclic shift operation to the W-MSA for extracting the features between patches. As illustrated by Figure 2. The Swin Transformer backbone network has four stages corresponding to four feature maps of different sizes. The feature map sizes are H/4 × W/4 × C, H/8 × W/8 × 2C, H/16 × W/16 × 4C, H/32 × W/32 × 8C. The feature map of each layer will be fed to the subsequent attentional FPN structure for further processing.

### Attentional FPN

Due to the high computational complexity of the Transformer architecture, which is of O(n2) level, only the feature map of the last layer (32 times down sampling) is utilized in the original DETR, resulting in the loss of small object feature information and has the limitation of single scale in feature representation. To address the above issues, we added the attentional FPN architecture to utilize all the features that output from the backbone network. The output of the first layer of the attentional FPN, which is also the last stage output from the backbone, is down sampled by 32 times. And then, for each neck level, the feature size is upsampled by 2 times. As the structure illustrated in Figure 2, the feature size of each neck level is 16 × 16, 32 × 32, and 64 × 64 when the input size is 512 × 512. Self-attention is aggregate to the FPN to focus on the regions of interest in each layer of the feature map. The feature map of each layer will be sliced into 8 times × 8 times pixels blocks, and the results computed between the blocks are used as weights for the output, the final computation is used to activate the part of interest in the feature map. Specifically, we add an auxiliary head after the attentional FPN to distinguish the foreground from the background, and only the foreground region will be input to the follow-up Transformer structure. The attentional FPN outputs the final blocks while recording the sparse encoding matrix of them, which is fed to the Transformer Decoder to map the features back to the original image.

Since each feature map is partitioned into 8 times × 8 times pixels blocks, there will be a total of 64 pixel blocks after the slice. Let’s define the input *x* = (*x*_1_, *x*_2_,…, *x*_64_). The input elements will be passed through an embedding layer W to obtain a multiset of one-dimensional vectors, denoted as *a* = (*a*_1_, *a*_2_,…, *a*_64_). Meanwhile, three learnable matrices are also set as *W^Q^*, *W^K^*, *W^V^*, represent the query matrix, the key matrix, and the value matrix, respectively. In particular for a single input *x*_*i*_, the proportion of its weights is calculated by the following formula:


(1) $$q_{i}=W^{Q}\,W\,x_{i}=W^{Q}\,a_{i}$$

(2) $$k_{i}=W^{K}\,W\,x_{i}=W^{K}\,a_{i}$$

(3) $$v_{i}=W^{V}\,W\,x_{i}=W^{V}\,a_{i}$$

(4) $$\alpha_{i,64}=\frac{q_{i}^{T}\cdot k_{i}}{\sqrt{d_{q,k}}},{\tilde{\alpha }}_{i,64}=S\,o\,f\,t\,m\,a\,x\,\left(\alpha_{i,64}\right)$$

(5) $$b_{i}=\sum_{i=1}^{64}{\tilde{\alpha }}_{i,64}\cdot v_{i}$$

The subscript (i, 64) of α in the above equation (4), represents that the similarity of the i-th input patch is currently being calculated, and there are a total of 64 patches to be calculated. The purpose of dividing by root *d*_*q, k*_ when calculating α is for normalization to avoid gradient vanish and *d*_*q, k*_ means the dimension of *q* and *k* vector. The softmax function is to map the sum of the weight ratios to 1, which is convenient for calculating *b*_*i*_.

### Transformer encoder-decoder

**FIGURE 3:**
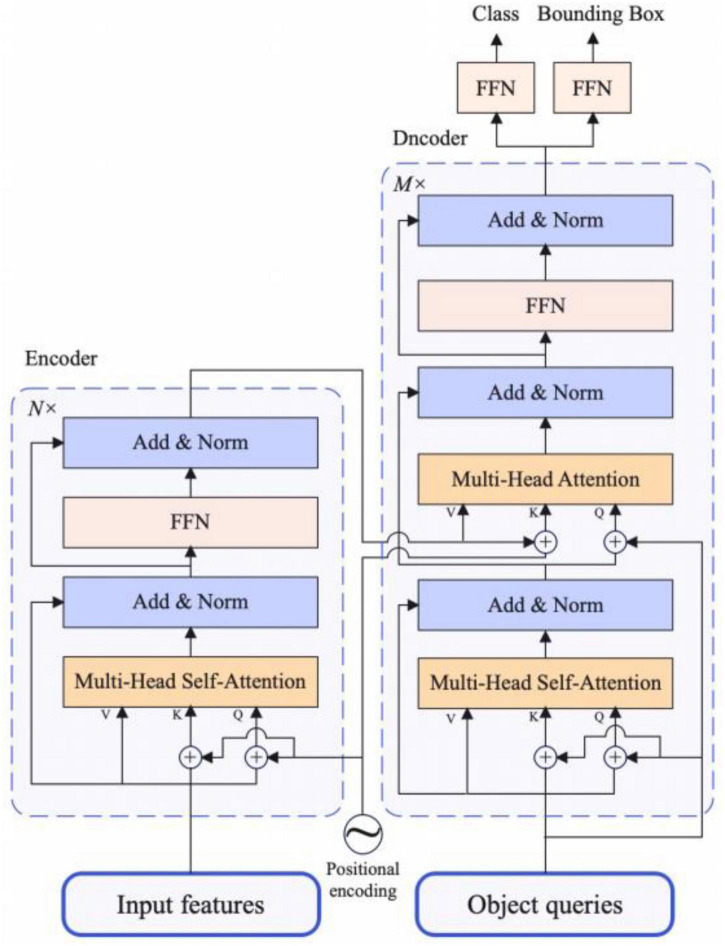
The architecture of transformer encoder and decoder.

The structure of Transformer encoder-decoder is shown in Figure 3, details are as follows:

#### Transformer encoder

The input requirement of Transformer encoder module is a sequence, so when getting the feature map output by attentional FPN, let the original feature map shape be C × H × W, we need to descend the channel dimension of the feature map first, and then flatten the H and W dimension. Finally, we get the feature map with the shape of C × L, where L equals to H × W. Each encoder block has a unified structure: multi-head self-attention, Add and Norm and feed forward network. As for Add and Norm structure, Add stands for residual connection to prevent network degradation, Norm is the layer normalization, which normalizes the activation values of each layer. Because of the invariance of Transformer Architecture, we add the fixed spatial positional encoding to the attention layer to complement the location information.

#### Transformer decoder

The architecture of the decoder part is the same as the traditional Transformer, but the difference is that each decoder module of our model decodes *N* inputs from encoder in parallel. Because the decoder structure is also permutation-invariant, the *N* inputs should be different so that different results can be generated. The meaning of object queries is similar to that of anchor in traditional object detection methods, and it is learnable. We input them to each multi-head self-attention module, which will eventually be decoded into object boxes location information and category information by the FFN structure, which is described in section “Detection head”.

To sum up, the features extracted from the backbone are passed through the multi-head self-attention module in the encoder structure along with the spatial positional encoding. After that, the *N* outputs of encoder and object queries are fed to the decoder part. Finally, the final object boxes and category information is output by the multiple multi-head self-attention and decoder-encoder attention and the FNN structure.

### Detection head

The role of detection head in DETR is to predicting a fixed set of object detections for each input image, rather than using a sliding window or anchor-based approach in traditional object detection models. After obtaining the output of the decoder, the final result is processed by a 3-layer perceptron and a linear projection layer, where the perceptron is comprized of a ReLU activation function. FNN computes the position of the box. The linear layer computes the category to which it belongs by softmax function. Since our predicted set is composed of *N* boxes, but in fact *N* is much larger than the actual number of objects present in the image, we mark the object query with no detected object as a background class. In particular, our FFN share the same weights and are calculated equally for all *N* object queries.

### Loss function

The loss function calculation is performed in two steps. First, finding the optimal pairwise loss between the ensemble prediction and the ground-truth label in the Hungarian algorithm alignment, the index of the set of solutions is set to $\hat{\sigma }$, as illustrated in formula (6), where *y_i_*is the set of prediction, ${\hat{y}}_{i}$is the ground-truth, both of them need to be stretched to a fixed length by adding None value, where length = max(len(*y*_*i*_), len(${\hat{y}}_{i}$)). The *L*_*MATCH*_ is defined as the gap between the predicted set and the ground-truth labels in the case of the first group pairing.


(6) $$\hat{\sigma }=a\,r\,g\,m\,i\,n\,\sum L_{M\,A\,T\,C\,H}\,\left(y_{i},{\hat{y}}_{i}\right).$$

Second, the index value $\hat{\sigma }$that calculated in the first steps is used to calculate the classification loss and the predicted bounding box loss, *c*_*i*_ is the class label, and the Hungarian loss is calculated as:


(7) $$L_{H\,u\,n\,g\,a\,r\,i\,a\,n}\,\left(\text{y},\hat{y}\right)=\sum_{i=1}^{N}\left[-l\,o\,g_{{\hat{p}}_{\hat{\sigma }\,\left(i\right)}}\,\left(c_{i}\right)+L_{b\,o\,x}\,\left(b_{i},{\hat{b}}_{\hat{\sigma }}\,\left(i\right)\right)\right]$$

where bounding box loss uses weighted IoU and L1 loss, *λ_*iou*_* and *λ_*L1*_* are the weight of IoU loss and L1 loss, respectively. The box loss is calculated as:


(8) $$L_{b\,o\,x}\,\left(b_{i},{\hat{b}}_{\hat{\sigma }}\,\left(i\right)\right)=\lambda_{i\,o\,u}\,L_{i\,o\,u}\,\left(b_{i},{\hat{b}}_{\hat{\sigma }}\,\left(i\right)\right)+\lambda_{L\,1}\,{||b_{i}-{\hat{b}}_{\hat{\sigma }}\,\left(i\right)||}_{1}$$

## Experimental results

### Dataset description

#### iChallenge-PM

Myopia has become a global public health burden. As the refractive error of myopia increases, high myopia will progress to pathological myopia, causing irreversible visual damage to the patient. Therefore, early diagnosis and regular follow-up are very important. With this challenge, the iChallenge competition jointly organized by Baidu Brain and Zhongshan Eye Center of Sun Yat-sen University provides iChallenge-PM, a dataset on pathological myopia, which includes 1,200 annotated retinal fundus images from non-pathological myopic subjects and pathological myopic patients (about 50%). There are 400 training data, validation data, and test data sets each.

#### Data augmentation

**FIGURE 4:**
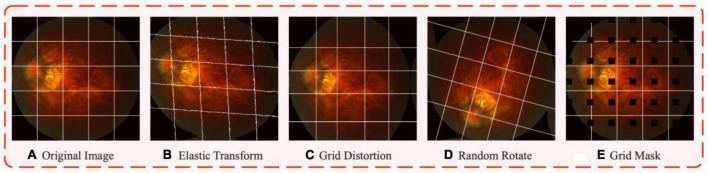
The visualized results after data augmentations. Subplot **(A)** is the sample of fundus images in iChallenge-PM dataset, the white grid line added to the original graph is to better show the effect of different augmentation. Subplot **(B–E)** represents elastic transform, grid distortion, random rotate, and grid mask, respectively.

We adopt several data augmentation methods to address the challenges of imaging angle and insufficient data. Subplot (b) in Figure 4 shows the elastic transform, which was proposed by Simard et al. (2003) and made great progress on the MNIST handwritten dataset, and the method is gradually applied to medical image processing and has been widely used [e.g., Mottl et al. (2002), Gulshad et al. (2021)], for the parameter settings we set the alpha to 50 and the sigma to 5. Subplot (c) is grid distortion, its effect is similar to that of the elastic transform, which is a non-rigid body transformation. Submap (d) is random rotation data augmentation, which aims to increase the diversity of imaging angles to solve the challenge of imaging angle, and the rotation angle is set from −180 to 180 degrees. Subplot (e) is the grid mask data augmentation proposed by Chen et al. (2020), which randomly masks a number of block locations on the image and fills them with 0 pixel values, this data augmentation can mask part of the positive samples with certain probability, thus preventing the model from overfitting to simple local features, for the parameter settings we set the ratio to 0.3 and the x_holes and y_holes to be set randomly between 5 and 7. The gains from each enhancement will be presented in the ablation study part.

### Evaluation metrics

For evaluating the performance of our detection method on iChallenge-PM dataset, we use mAP as the evaluation metrics, which is the mean average precision of all categories. AP θ is calculated as the area enclosed by the Precision (P) and Recall (R) and the coordinate axis at an IoU (Intersection over Union of predicted box and ground-truth) threshold of θ, as illustrated below:


(9) $$I\,o\,U\,\left(A,B\right)=\frac{A\,⋂B}{A\,⋃B}$$

(10) $$P=\frac{T\,P}{T\,P+F\,P}$$

(11) $$R=\frac{T\,P}{T\,P+F\,N}$$

where TP (True Positive) is the number of IoU between the predicted box and the ground-truth label that is greater than or equal to the threshold θ, while FP (False Positive) means the number of IoU between the predicted box and the ground-truth label that is less than the threshold θ. FN (False Negative) means no positive object is detected. Finally, we can get mAP by averaging AP θ at different thresholds.

### Implementation details

Our model is implemented using MMDetection object detection algorithm library based on Pytorch1.8 deep learning framework using four NVIDIA RTX 3090 GPUs. We pre-trained our model on COCO dataset for 36 epochs and fine-tuned on iChallenge-PM for 100 epochs with a mini-batch size of 16 due to the limited data amount. The learning rate is initiated to 0.001, and we use CosineAnnealingLR to decay the learning rate with 5 epochs warm-up. The AdamW optimizer is used to optimize the hyper-parameters. For evaluating model performance, the IoU threshold and confidence threshold are set to 0.5 and 0.01, respectively. NMS is adopted as post-process method. As for the data augmentation implementation approach, we adopt the image processing algorithms based on opencv-python and Albumentations library (Buslaev et al., 2020).

### Ablation study

To verify the effectiveness of our attentional FPN, we set up a set of ablation experiments. The backbone network used as the base model was Swin-Small, DETR and its variants are selected to test the gains we obtained by adding attentional FPN. In object detection, taking COCO object definition as an example (Kisantal et al., 2019), we define a small object as an object which pixel number is less than 32 × 32, a medium object is an object which pixel number is between 32*32 and 96*96, and a large object is an object which pixel number is larger than 96*96. AP_S_ means object area smaller than 32 × 32 pixel points, AP_M_ means object area between 32 × 32 and 96 × 96 pixel points, AP_L_ means object area bigger than 96 × 96 pixel points. As can be seen from Table 1, the DETR works well for large target detection, but suffers from a major shortcoming in small object detection. If the FPN is added directly to the DETR structure, since the Tranformer has an O(n^2^) computational complexity and limited computational resources, this method will result in an out of memory (OOM) error. In contrast, by adding a self-attention mechanism to the FPN, only a small number of features need to be fed into the Transformer structure, and our model achieves 29.02% improvement in detecting small objects, and obtains 8.19 and 1.53% improvement in detecting medium and large object, respectively. It is worth noting that Deformable DETR, Conditional DETR and our method achieve more significant improvement with multi-scale (MS) than the other models with single-scale (SS) design. The result indicates that multi-scale learning allows greater variety of features. With Attentional FPN, our model not only achieves a significant improvement in capturing small objects, but also a significant improvement in convergence speed during training. In a nutshell, our model not only achieves a significant improvement in capturing small objects, but also a significant improvement in convergence speed during training. Note that all experiments adopt the same data augmentation methods.

**TABLE 1:** The effects of attentional FPN.

Model	AP_50_	AP_S_	AP_M_	AP_L_	Epochs	FPS	MS/SS
DETR 120e	62.53	29.52	65.93	96.18	120	19.31	SS
DETR 200e	71.21	39.12	74.89	97.62	200	19.31	SS
DETR 300e	71.87	39.69	75.47	97.81	300	19.31	SS
DETR + FPN	OOM	–	–	–	–	–	SS
UP-DETR	76.08	44.13	81.77	98.03	300	16.21	SS
Deformable DETR	80.53	58.92	82.55	98.76	120	17.86	MS
Conditional DETR	84.29	65.35	**83.73**	99.10	120	16.60	MS
Ours	**85.18**	**68.71**	83.66	**99.34**	120	8.73	MS

Bold values represent the best metric values achieved by our method and other comparison methods.

Furthermore, it is important to carefully evaluate the impact of data augmentation on model performance, which can provide insights into the factors that contribute to model performance. In Table 2, we focus on the AP boost from the augmentation method, presenting in a cumulative manner. As can be seen evidently, Random Rotate brings the most significant performance gains because it simulates a situation that is similar to the characteristics of the original data (i.e., the lesion sites tend to appear in different directions), this greatly expands the training sample. The other augmentation methods also achieved considerable improvements, proving that the increment of data volume by the data augmentation methods is effective to improving model performance.

**TABLE 2:** Performance improvement on various augmentations.

Original image	Elastic transform	Grid distortion	Random rotate	Grid mask	AP_50_
√					80.45
√	√				81.81
√	√	√			82.33
√	√	√	√		85.11
√	√	√	√	√	**85.18**

Bold values represent the best metric values achieved by our method and other comparison methods.

### Comparison study

**FIGURE 5:**
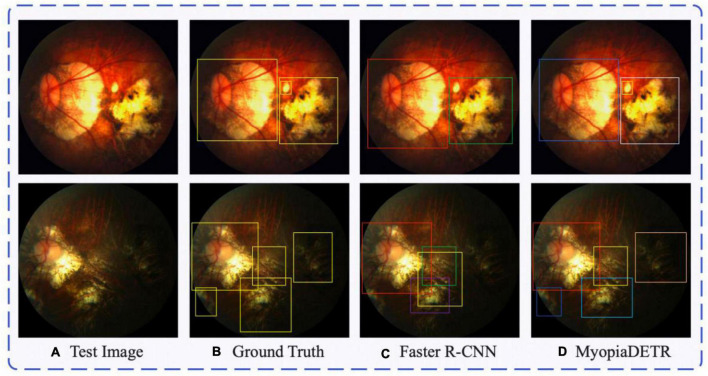
The comparison between traditional object detection algorithm Faster R-CNN and MyopiaDETR. Subplot **(A)** shows the test image, subplot **(B)** shows the PM location labels, subplot **(C)** shows the output of the Faster R-CNN algorithm, and subplot **(D)** shows the output of our MyopiaDETR.

Subplot (a) in Figure 5 shows the test image, subplot (b) shows the PM location labels, subplot (c) shows the output of the Faster R-CNN algorithm, and subplot (d) shows the output of our MyopiaDETR. The detection result in the first row of subplot (c) fails to detect the small object compared to the ground truth, and the traditional object detection algorithm has limited learning of complex morphological features. The yellow box in the second row of subplot (c) is the model’s false detection, and if the threshold of post-processing of NMS is adjusted down, it will lead to the purple or green box being removed by the post-processing algorithm, and the detection ability is still very poor for irregular PM regions. Our MyopiaDETR does not have these problems, and not only can handle the detection of irregular PM, but also do not have to worry about the false removal of detection boxes caused by post-processing.

In addition, we compare the effect of different backbone networks on the performance of our model. As shown in Table 3, in the small to medium sized network architecture with a similar number of parameters, Swin Transformer shows better performance, we believe this is because the feature extracted by Swin Transformer is consistent with the operations in attentional FPN. Due to the morphology irregularity, we need a feature extraction network with strong capability to capturing global context feature. Swin Transformer has better feature extraction capability than ResNet because of its global field of view. Thus, the Swin Transformer avoids the problem of morphology irregularity and shows a better detection performance than the ResNet. However, because the Transformer architecture lacks inductive bias, as Swin Transformer becomes deeper, it requires a lot more data and the model performance is somewhat weakened. Compared to the CNN structure, the global feature representation capability of Transformer is more prominent, and Swin Transformer performance has a significant advantage over the CNN structure in the case of small model structures.

**TABLE 3:** Comparisons between different backbones.

Backbone	AP_50_	AP_S_	AP_M_	AP_L_
Swin-S	85.18	68.71	83.66	99.34
Swin-B	86.23	69.76	84.65	99.35
Swin-L	85.62	69.08	83.72	99.34
ResNet-50	82.25	65.91	80.34	98.72
ResNet-101	84.67	67.21	82.76	99.12
ResNet-152	86.32	69.65	84.78	99.21

**FIGURE 6:**
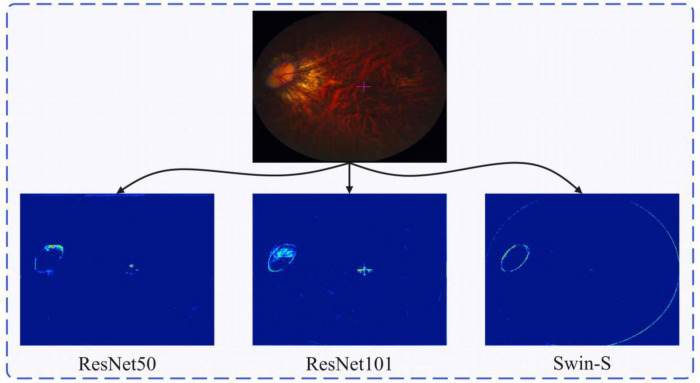
Retinal fundus image and the corresponding feature heatmap generated by different backbones, the heatmap of the feature learned by ResNet50, ResNet101, and Swin-S from left to right.

Figure 6 shows the 8 × down sampled feature maps of ResNet-50, ResNet-101, and Swin-S. As the CNN is more localized, it will to some extent activate non-focal regions, such as the cross-focused symbols in the figure. The Swin Transformer structure, on the other hand, has a global field of view and can focus on more important information (e.g., lesion borders as well as slice edge contours). As shown in Figure 6, Swin Transformer has a better understanding of the global information and can pay more attention to the global contour information. ResNet, on the other hand, has a strong feature extraction capability, but it only pays attention to the local contour information and has more activation within the local contour information. In contrast, we do not need to pay attention to all the information within the local contour information when dealing with the ophthalmic disease region, thus highlighting the superiority of Swin Transformer in feature extraction.

## Discussion

The sources of novelty in our work are: (1) Ordinary deep learning-based object detection methods utilize many modules with obvious traces of artificial design, that is, a paradigm that does not match the way human vision finds objects. (2) DETR proposes a new object detection paradigm based on ensemble prediction, which directly predicts all objects in an image without post-processing, which is more in line with human visual habits. (3) Due to the high computational complexity of Transformer and the fact that DETR only uses the output features of the last layer of the model, which contains rich semantic information but is weak in the representation of local information, the performance in small object detection is poor, so we propose attentional FPN for feature aggregation, which uses all the output features of the backbone network, significantly improve the performance in small object detection.

However, although our proposed new method solves the problem of small object detection as well as purifying the background features, there are still some drawbacks. The use of Swin Transformer as backbone makes the training time much longer than traditional DETR model. Specifically, in DETR, each query is responsible for a part of the location and size of the object in an image, which requires all objects in all images in the training set to be well apportioned to different queries, so more epochs are needed. The data amount is very important for deep Transformer architecture model. Our follow-up research is considering to replace the backbone network with an optimized architecture to adapt to scenarios with small data volumes.

## Conclusion

In this paper, we propose attentional FPN and use a new paradigm for object detection to solve the eye disease detection challenges based on 2D fundus images. The experimental results show that our attentional FPN can be adapted to other deep learning architectures with only a small increment in computational cost to achieve a significant accuracy improvement. Several augmentation methods are utilized to improve the data volume and make our model achieve considerable performance improvements, proving that the increment of data volume by the data augmentation methods is effective to detecting lesion area in 2D fundus images. Our model not only achieves a significant improvement in capturing small objects, but also a significant improvement in convergence speed during training.

## Data availability statement

Publicly available datasets were analyzed in this study. This data can be found here: https://aistudio.baidu.com/aistudio/datasetdetail/177172.

## Author contributions

ML, SL, and ZJW contributed to the statistical analyses and wrote the manuscript. ZHW, ZY, and XL contributed to the data collection and figures plot. RZ provided the technique and wrote the guidance. All authors read and approved the final manuscript, contributed to data collection and article, and approved the submitted version.
